# Supplementary material for: Flight and Reproduction Variations of Rice Leaf Roller, Cnaphalocrocis medinalis in Response to Different Rearing Temperatures
Source: Insects. 2021 Dec 2;12(12):1083. doi: 10.3390/insects12121083 (PMC8706861; doi:10.3390/insects12121083)
Supplement: Supplementary file 1 [file insects-12-01083-s001.zip › insects-1486683-supplementary.pdf]

Supplementary Table S1. Reproductive performance of *C. medinalis* females between the flying and non-flying groups treated by different rearing temperatures during the immature stage

Parameters	Treatments	Rearing temperatures			
		18 °C	22 °C	26 °C	34 °C
Preoviposition period (d)	Flying	6.88 ± 0.49a **	6.32 ± 0.71a	5.61 ± 0.35a*	5.30 ± 0.30 a**
	Control	4.91 ± 0.42 b	5.12 ± 0.26 b	7.97 ± 0.90 a	7.38 ± 0.48 a
Period of first oviposition (d)	Flying	3.88 ± 0.49 ab	4.32 ± 0.71a	2.61 ± 0.35 ab	2.30 ± 0.30 b**
	Control	2.91 ± 0.42 a	3.12 ± 0.26 a	3.97 ± 0.90 a	4.38 ± 0.48 a
Lifetime fecundity	Flying	94.75 ± 16.34 a**	46.16 ± 10.48 ab**	78.30±17.70 ab	30.35 ± 8.61 b
	Control	214.23 ± 28.31 a	181.40 ± 20.13 a	103.20 ± 17.38 b	74.62 ± 20.83 b
Mating frequency	Flying	0.33 ± 0.10 a**	0.44 ± 0.10 a*	0.48 ± 0.11 a	0.30 ± 0.10 a
	Control	1.05 ± 0.20 a	0.81 ± 0.10 ab	0.51 ± 0.08 bc	0.42 ± 0.10 bc
Female longevity (d)	Flying	15.21 ± 1.07 a	9.44 ± 0.89 b**	11.96 ± 1.05 ab**	9.13 ± 0.55 b
	Control	16.27 ± 0.93 a	13.74 ± 0.82 a	15.54 ± 0.68 a	10.58 ± 0.97 b
Oviposition period (d)	Flying	7.92 ± 0.75 a	3.72 ± 0.63 b**	5.56 ± 0.95 ab	3.26 ± 0.44 b
	Nonflying	8.05 ± 0.98 a	7.76 ± 0.72 a	6.74 ± 0.79 ab	4.04 ± 0.63 b
Mating percentage (%)	Flying	33.33 ± 9.83 a*	44.00 ± 10.13 a*	47.83 ± 10.65 a	30.43 ± 9.81 a
	Control	68.18 ± 10.16 a	66.67 ± 7.36 a	51.43± 8.57 a	41.67 ± 10.28 a

Different lowercase letters in the same row indicate significant differences by Tukey's HSD test at 5% level. "*", "**" represent significant or highly significant differences in the same column by t-test (p <0.05 or p <0.01). Sample sizes for each flying treatment are 22, 42, 35, 22 and 24 females, and each control treatment are 24, 25, 23, 15 and 23 females, from left to right, respectively.
